# Preparation of Cementitious Materials from Mechanochemically Modified Copper Smelting Slag Compounded with High-Aluminum Fly Ash

**DOI:** 10.3390/ma17030546

**Published:** 2024-01-23

**Authors:** Dige Sheng, Jirong Lan, Zhengyu Du, Yantao Ma, Min Zhou, Haobo Hou

**Affiliations:** 1 School of Resource and Environmental Sciences, Wuhan University, Wuhan 430072, China; 2 Hubei Environmental Remediation Material Engineering Technology Research Center, Wuhan 430072, China; 3 Central Southern China Electric Power Design Institute Co., Ltd. of China Power Engineering Consulting Group, Wuhan 430071, China

**Keywords:** copper smelting slag, high-aluminum fly ash, mechanical activate, cementitious materials

## Abstract

Copper smelting slag discharged from mining and high-aluminum fly ash generated during the combustion of coal for energy production are two typical bulk solid wastes, which are necessary to carry out harmless and resourceful treatment. This research proposed an eco-friendly and economical method for the co-consumption of copper smelting slag and high-aluminum fly ash. Cementitious materials were compounded with copper smelting slag and high-aluminum fly ash as the main materials were successfully prepared, with a 28-d compressive strength up to 31.22 MPa, and the heavy metal leaching toxicity was below the limits of the relevant standards. The optimum mechanical properties of the cementitious materials were obtained by altering the material proportion, ball mill rotation speed, and CaO dosage. Under the combined effect of mechanical ball milling at a suitable speed and chemical activation with a certain alkali concentration, the prepared cementitious materials had an initial activation. The pastes of the cementitious materials generated a gel system during the subsequent hydration process. The two steps together improved the mechanical strength of the cured products. The preparation was simple to operate and offered a high stability of heavy metals. The heavy metal contaminants were kept at a low content throughout the process from raw materials to the prepared cured specimens, which was suitable for application in practical environmental remediation projects and could provide effective solutions for ecological environment construction.

## 1. Introduction

Copper mining is part of the world’s most important mining industries. According to the USGS, approx. 22 million tons of copper was extracted across the world in 2022, and copper reserves stood at approximately 890 million tons [1]. The growth of the world’s population and the wider application of copper and copper allied products in the electrical, construction, and telecommunication industries will intensify the high demands for copper. Therefore, even lower grade ores with a high waste volume output will be processed to satisfy these growing demands [2]. Significant amounts of mine wastes, including mine waste rock (a product from the ore extraction stage) and tailings (generated during ore processing), are generated by exploiting deposits to produce ore with an economic value [3]. Tailings discharges will continue to grow as copper ore grades continue to decline. Due to its chemical (e.g., acid mine drainage) and physical risks (e.g., dam failure), the mining industry has been widely criticized for its negative environmental impacts [4]. At present, a small number of tailings can be used as filler in waste ore but the vast majority can only be stored in engineered dams and impoundments [5]. The recovery of valuable substances from copper mine wastes has attracted extensive attention due to the high economic benefits, such as the recovery of copper from copper slag [6,7], and the extraction of other metals (e.g., iron, zinc, and cobalt) from slag [8,9]. Copper smelting slag had also been utilized in concrete areas because of its similar chemical composition with common construction materials. Copper slag possesses the basic conditions for creating hydrated materials and provides a great substitute for natural sand as an aggregate [10,11]. Lori et al. prepared concrete with a best 28-d compressive strength of 23.45 MPa by replacing 60% dolomite with copper slag as a coarse aggregate [12]. Rohini et al. revealed that replacing 50% to 75% of the sand with copper slag produced concrete with superior mechanical properties when treated with microorganisms [13]. Ambily et al. prepared ultra-high-performance concrete (UHPC) by completely replacing standard sand with copper slag and achieved a 28-d compressive strength of 162 MPa, which was only 15% lower than the samples with standard sand [14]. The above research used copper slag as an aggregate, partially or completely replacing the conventional construction materials. They all resulted in excellent 28-d compressive strengths, but none of them could be separated from cement as the main material.

Another industrial solid waste representing a challenge to be solved is coal fly ash, generated during the combustion of coal for energy production. In Shanxi, the major coal and electricity province of China, fly ash production remains high and most of it can only be stored in the wild due to limited capacity and insufficient market demand. In Shuozhou city alone, 30 million tons of fly ash continue to be generated annually from an existing inventory of nearly 150 million tons. The improper disposal of fly ash presents a variety of environmental problems, thus considerable research has been undertaken on the subject worldwide for years. The major utilization of fly ash has been in construction, such as mine back filling, light weight aggregates, road sub-bases [15], and green concrete [16]. The American Society of Testing Materials divided fly ash into Class C and Class F ash based on the oxide content [17]. An advanced method using automated SEM was applied to present the physical and chemical characteristics of fly ash [18]. The high-aluminum fly ash (HAFA) contained lower SiO_2_ and higher Al_2_O_3_, which distinguished HAFA from the traditional classification. Considering this feature, comprehensive applications of HAFA have come to light, such as in the preparation of high-performance ceramics [19,20,21], the preparation of mesoporous alumina adsorbents for the removal of heavy metals from water [22], and the extraction of valuable materials such as Al, Li, and Ga [23].

Mechanochemistry synthesis is an effective method for the preparation of multi-component crystalline systems, which was first defined by Ostwald in 1919. As an inexpensive and simple process, mechanochemistry synthesis was widely used in reactions between various media to synthesize a wide range of materials [24]. It provided repeated fracturing to break up particles, decrease the crystallite size, introduce surface defects, and cause other physical changes [25]. High-speed ball milling allowed two or more components to co-crystallize and change their crystal structure to achieve activation, which often resulted in unsaturated groups, free ions, and electrons to promote some chemical reactions [26,27].

Although the above solid waste treatment and disposal technologies could alleviate the problem of large stockpiles of copper smelting slag and high-alumina fly ash, these technologies either require calcination, which increases energy consumption and carbon emissions, or involve complex processes and a high equipment investment, thus a key technology with low energy consumption and a low investment for solid waste disposal is desperately required. This paper aimed to achieve the simultaneous abatement of two solid wastes by the preparation of composite cementitious materials. Innovatively, the cementitious materials were prepared using only two solid wastes, copper smelting slag and high-aluminum fly ash, without cement. The prepared cementitious materials had creditable mechanical properties with a 28-day compressive strength up to 31.22 MPa, and the leaching concentration of heavy metal pollutants was beneath the relevant standard limit. The whole preparation process was low-cost, easy to manipulate, and environmentally friendly with a good stability of heavy metals. The product can be utilized in practical environmental restoration projects, such as road foundations, lagoon closures, and quarry restoration, which provide efficient solutions for ecological environment construction.

## 2. Materials and Methods

### 2.1. Raw Materials

The copper smelting slag (CMS) used in the study was provided from a copper smelting plant in Hubei Province, China. The high-aluminum fly ash (HAFA) was from a coal-fired power plant in Shanxi Province, China.

### 2.2. Ball Milling Treatment

A planetary ball mill (TianChuang, Changsha, China) and two ceramic milling cylinders (500 mL) were used. The mixture samples and three times weight ceramic balls (the ratio of the spheres was Φ2 mm:Φ5 mm:Φ10 mm = 2:2:1) were placed into the ceramic containers up to about a two-thirds capacity. The milling was rotated horizontally at six different milling speeds for the same period (1 h). The rotational direction of the ball mill was changed every 15 min. The resulting samples were sealed for the next analysis.

### 2.3. Preparation of the Cementitious Materials

**Figure 1:**
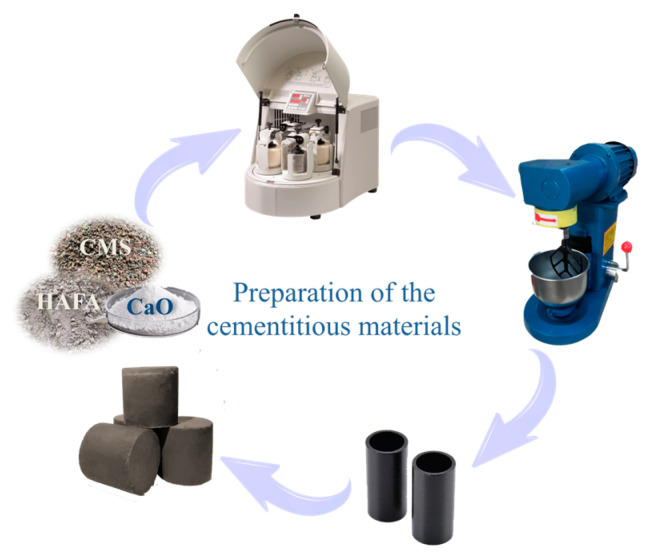
Flow chart of the preparation.

The two raw materials were dried to a constant weight at 75 °C, mixed with calcium oxide (CaO) at a certain ratio after cooling and sieving, then activated by mechanical ball milling. In this study, the material proportion, ball mill rotation speed, and CaO dosage were selected as the objects of study for the single-factor experiments. The test design scheme is shown in Table 1. The cementitious materials were mixed with distilled water in a proportion of 25 wt% using a cement paste mixer, and the pastes were poured into a cylindrical cast iron experimental mold. Later, the materials were molded using a die-casting machine under 40 kN of pressure and then demolded on a hydraulic electric stripper. The samples were cured in a standard curing cabinet at 25 °C and 95% humidity until the specified curing ages of 7 d, 14 d, and 28 d. Figure 1 illustrates the flow chart of the preparation.

### 2.4. Mechanical Property Test

The compressive strength was determined using cylindrical specimens (Φ 50 mm × 50 mm), and each investigated composition was tested three times. The mechanical strength of the specimens was measured using a universal material testing machine (WE-300 S, Shaoxing, China) in accordance with the Chinese standard (JTG E51-2009) [28]. The strain rate of the compressive strength tests was maintained at 1 mm/min.

### 2.5. Heavy Metals Leaching Experiment

According to the “Solid Waste Extraction Procedure for Leaching Toxicity–Acetic Acid Buffer Solution Method” (HJ/T 300-2007) [29], a mixed solution of acetic acid and sodium hydroxide was used to prepare the leaching agent with a pH of 4.93 ± 0.05 for the leaching toxicity test. The samples were the powder of CMS, HAFA, and a set of 28-d cured specimens. The specimens were mixed with the acid solution at a ratio of 1:20 and shaken for 18 h (overnight) at a speed of 30 rpm with a tilting shaking device. After completing the shaking, the supernatant was filtered using a disposable sterile syringe with a polyethersulfone (PES) membrane filter (0.22 μm). Blank extraction was performed simultaneously throughout the process. The heavy metal ion concentrations in the leachate were determined using an inductively coupled plasma optical emission spectrometer (ICP-OES). The leaching toxicity of the heavy metal ions was referred to the standard limits of various heavy metals specified in IV-class water from the national Chinese standards of GB 3838-2002 [30] and GB/T 14848-2017 [31,32].

### 2.6. Chemical and Mineralogical Characterization

The elemental composition of the CMS and HAFA was determined using X-ray fluorescence spectrometry (XRF, Thermo Scientific, ARL Perform’X, Waltham, MA, USA). The particle size of the two raw materials and powder with different ball mill rotation speeds was measured using laser granulometry (Malvern, Mastersizer 2000, Worcestershire, UK). The mineralogical composition was analyzed using X-ray diffraction (XRD, Rigaku, MiniFlex 600, Tokyo, Japan). The atomic valence states of the materials were detected using X-ray photoelectron spectroscopy (XPS, Thermo Scientific, K-Alpha, Waltham, MA, USA). The microstructures of the samples were investigated using a field emission scanning electron microscope (FE-SEM, Zeiss, Sigma 300, Oberkochen, Germany). ICP-OES (PerkinElmer, Avio 200, Waltham, MA, USA) was used to determine the heavy metal leaching species and the content of raw materials and cured specimens.

## 3. Results and Discussion

### 3.1. Physical and Mechanical Properties

#### 3.1.1. Particle Size

**Figure 2:**
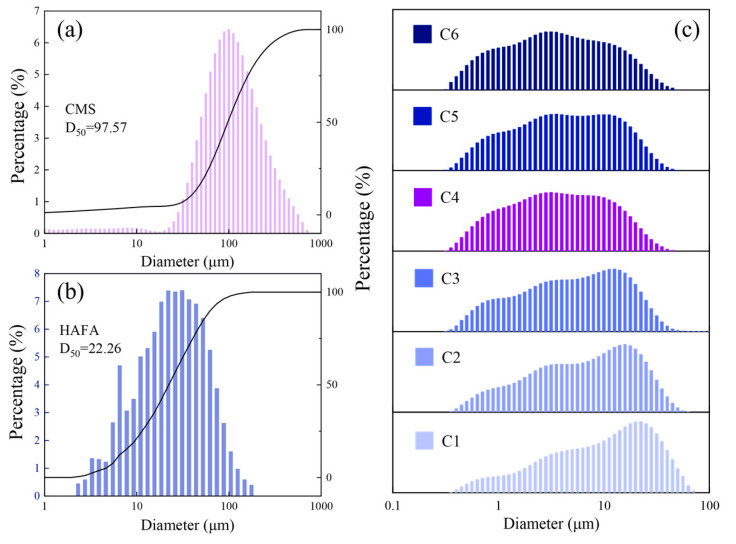
Particle size distribution of the raw materials (**a**,**b**) and blended mill products (**c**).

In cementitious material systems, particle size distribution is an essential indicator for examining the cementing activity of the mixture [33]. Within a certain fineness range, different particle sizes of fly ash could produce differences in the macroscopic properties of the cementitious material [33,34]. Figure 2a,b shows the results of the particle size distribution of the two raw materials, the D50 of the CMS and HAFA were 97.57 μm and 22.26 μm, respectively, and Figure 2c shows the result of the blended materials (CMS, HAFA, CaO) after 1 h of ball milling at six different rotation speeds. The main particle size results of all the materials are shown in Table 2, it was clear that the D50 and D90 of the mechanically ball milled product decreased significantly with respect to the raw material without ball milling, indicating that mechanical ball milling treatment could reduce the particle size of the raw materials mixture sufficiently and effectively. As the rotation speed increased from 350 rpm to 650 rpm, the particle size of the ball milled product showed an overall decreasing trend, which demonstrated that different rotation speeds had an effect on the particle size of the ball milled product. Another promising finding was that the particle size of C5 (D50 = 4.04 μm, D90 = 16.36 μm) increased compared to C4 and C6, confirming that the materials achieved initial self-gelling at 600 rpm. The comparisons shown in Figure 2c revealed that the particle size distribution peaked in the range of 1–10 μm for the samples with speeds above 550 rpm, indicating that mechanical ball milling could convert mechanical energy into internal energy to break the particle structure, which could become a significant method for stimulating the cementing activity of the ball milled mixture [35].

#### 3.1.2. Compressive Strength

**Figure 3:**
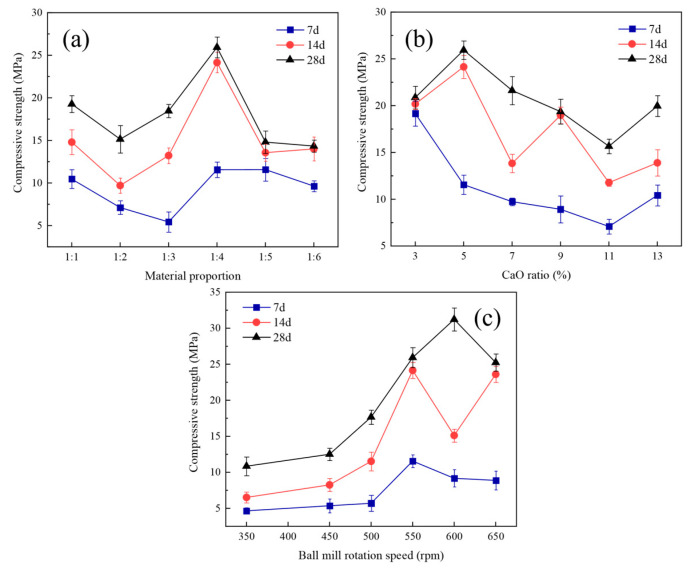
Variation trend of the compressive strength of the cured specimens at different ages with the material proportion (**a**), CaO dosage (**b**) and rotation speed (**c**).

The results of the single-factor experiments on the compressive strength are shown in Figure 3. The raw material proportion, CaO dosage, and ball mill rotation speed all affected the compressive strength of the cementitious materials. As shown in Figure 3a, the compressive strength of the cured specimens peaked at 1:4 for the CMS: HAFA (25.93 MPa) as the material proportion varied for a certain percentage of CaO (5%) and the ball milling speed (550 rpm). The particles of the CMS were coarse (D50 = 97.6 μm), while the particles of the HAFA were fine (D50 = 22.3 μm), which contained micro-beads and debris of very small particle sizes, corresponding to active nanomaterials. With the gradual increase in the proportion of HAFA, the fine-grained aggregate filled the voids between the coarse aggregates, producing a microaggregate effect that improved the homogeneity and denseness of the cementitious material significantly. However, the excessive content of the HAFA led to an uneven distribution of contact surfaces between the coarse and fine particles, and the spare fine aggregate had no place to combine with the coarse aggregate, which in turn reduced the structural strength and mechanical properties of the cementitious material.

As shown in Figure 3b, the compressive strength of the cured specimens showed a general trend of increasing and then decreasing with the increase in CaO addition, reaching a maximum at 5% when the material proportion (1:4) and rotation speed (550 rpm) were certain. The result above was attributed to the “concentration effect”, where the strength of the cementation system improved as the concentration of CaO increased from 3% to 5%, with a gradual growth in the concentration of OH- and an acceleration of the hydration reaction rate. However, when the concentration exceeded 5%, the rate of the slag structure destruction and hydration product formation was too fast, and the hydration products lacked time to diffuse and form a protective film on the surface of the slag particles, hindering the later hydration reaction process and leading to a reduction in strength [36]. As shown in Figure 3c, the compressive strength of the cured specimens increased and then decreased as the ball milling speed gradually increased from 350 rpm to 650 rpm, reaching a peak (31.22 MPa) at the speed of 600 rpm. The mechanical activation was achieved by ball milling in this experiment. During the milling process, it was found that the cured specimens at speeds below 500 rpm were relatively soft, and the compressive strength was significantly different with the cured specimens at a higher speed, suggesting the failure of activation. By combining the compressive strength with the particle size results, it was found that the peak of the compressive strength at 600 rpm was probably due to the formation of tiny agglomerates in the ball milled mixture at this speed, which led to an increase in the strength of the subsequent hydration product.

The average value of the 28-d compressive strengths were higher than 15 MPa and were considered for the preparation of MU15 bricks, which was referred to the national Chinese standard for solid concrete brick (GB/T 21144-2023 [37]).

### 3.2. Chemical Properties

#### 3.2.1. XRF Analysis

The main chemical composition of the copper smelting slag was CaO, SiO_2_, Fe_2_O_3_, and MgO. The main chemical composition of the HAFA was SiO_2_, Al_2_O_3_, and CaO (Table 3). The contents of the trace elements of the two raw materials are shown in Table 4, showing that the levels of the major heavy metal elements in both materials were extremely low. To further determine the non-toxicity of the materials, toxic leaching experiments were subsequently performed on both the raw materials and the cured specimens.

#### 3.2.2. XRD of the Materials

**Figure 4:**
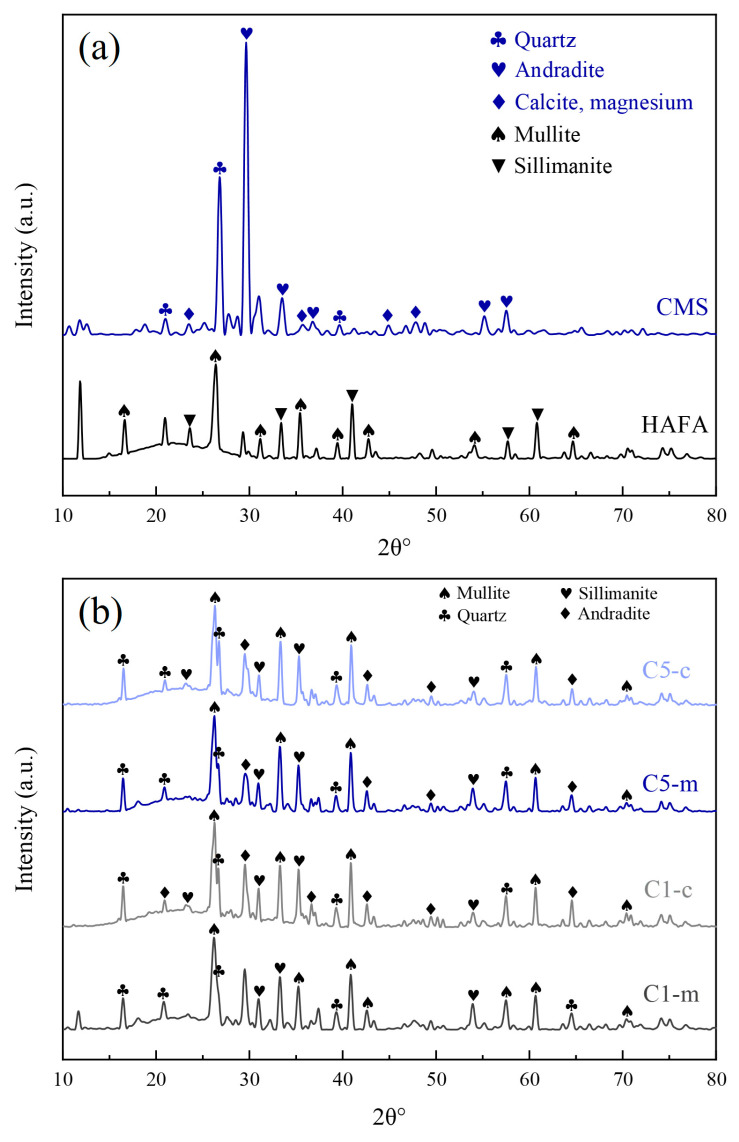
XRD analysis of the raw materials (**a**) and materials after milling and curing (**b**).

Based on the mechanical property analysis, the best macroscopic properties of the cured specimens were obtained using sample C5 as the precursor, while C1, which had the worst mechanical properties, was selected as the control group. The diffraction pattern of the raw materials is shown in Figure 4a and the results of C1-m, C1-c, C5-m and C5-c are shown in Figure 4b. Here “-m” represents the materials after ball milling (before curing), and “-c” represents the 28-d cured specimens (after curing). The crystalline phases of the CMS were mainly quartz (PDF#87-2096), andradite (PDF#84-1937), and calcite (PDF#89-1305). The main components in the HAFA were mullite (PDF#74-2419) and sillimanite (PDF#88-0891), and the broad hump between 20–25° was due to amorphous silica–aluminate vitreous structures, which indicated that the HAFA was prone to pozzolanic reactions during the hydration process. After ball milling, the two raw materials were completely mixed and the main composition of the product was not drastically changed, with the appearance of the diffraction peaks belonging partly to mullite and quartz, where the minerals were apparently derived from fly ash and CMS, respectively. Calcite in the CMS was converted to amorphous calcium carbonate by external forces from ball milling, which exhibited a broad hump from 20° to 35° on the XRD pattern [38]. The diffraction pattern of the materials after ball milling and 28-d curing also exhibited broad humps at around 20–25°, with the greater intensity of the broad peak representing a higher content of amorphous materials. In the cured samples, the diffraction patterns had more intense broad humps, indicating that a new amorphous phase was produced after hydration, which was also confirmed by the SEM results. These amorphous phases were the result of the mechanical activation, and the amorphous layer was then able to react with Ca(OH)_2_ deriving from the hydration of CaO [39].

#### 3.2.3. SEM Images

**Figure 5:**
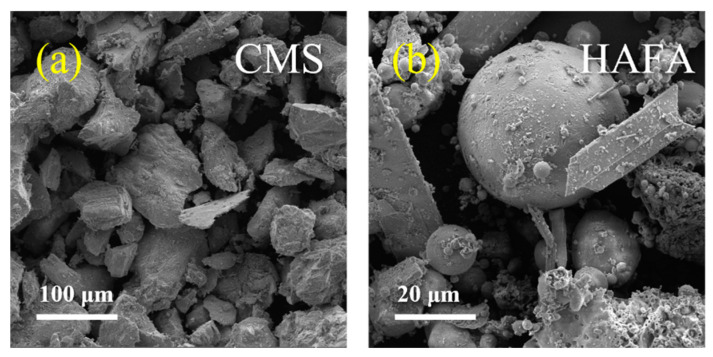
SEM images of the CMS (**a**) and HAFA (**b**).

**Figure 6:**
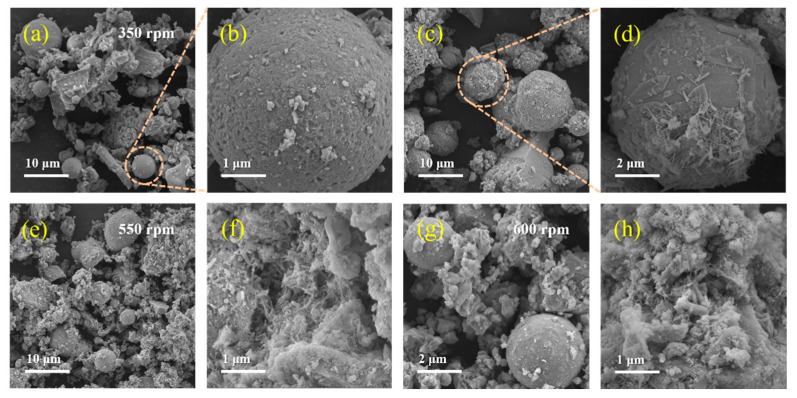
SEM images of C1-m (**a**,**b**), C1-c (**c**,**d**), C4-m (**e**), C4-c (**f**), C5-m (**g**), and C5-c (**h**).

The SEM images of the raw materials are shown in Figure 5a,b. The images of C1-m, C1-c, C4-m, C4-c, C5-m and C5-c are shown in Figure 6a–h, respectively. The CMS particles were dominated by irregular, lumpy, and a few flaky particles with large particle sizes. The HAFA particles were mainly spherical, columnar, and other irregular structures with smaller particle sizes. After 1 h of ball milling at 350 rpm, the large structures in the raw materials were broken into smaller pieces, while the particles were loosely bound to each other. The cured products under a speed of 350 rpm still retained its original spherical and lumpy shape. The HAFA was not activated and there was no massive agglomeration between the particles. The previously smooth surface of the spheres in the HAFA was roughened to facilitate the adhesion of some hydration products. Combined with the XRD results, the needle-like columnar crystals attached to the spheres were presumably the product of the regeneration of sillimanite after a reaction with the other precursor materials. The increase in the rotation speed apparently contributed to more complete fragmentation and small particle agglomerations of the ball mill product. The cured products at 600 rpm hardly observed the spherical characteristic material of the fly ash, indicating that the HAFA was completely activated into a generate amorphous cementitious substance. By combining the SEM images with the XPS spectrum, it was found that SiO_2_ and Al_2_O_3_ in the raw materials were gradually dissolved by OH^−^, the Si–O and Al–O bonds were broken, and the silicates and meta-aluminates were continuously dissolved and spread between the particles, reconstituting an unstable silica–aluminate system, which polymerized at room temperature to form a three-dimensional mesh of the cementitious material that encapsulated the surrounding particles. These structures filled the pores of the cured specimens, forming a morphologically stable and structurally compact cementitious system.

#### 3.2.4. XPS Spectrum of the Materials

**Figure 7:**
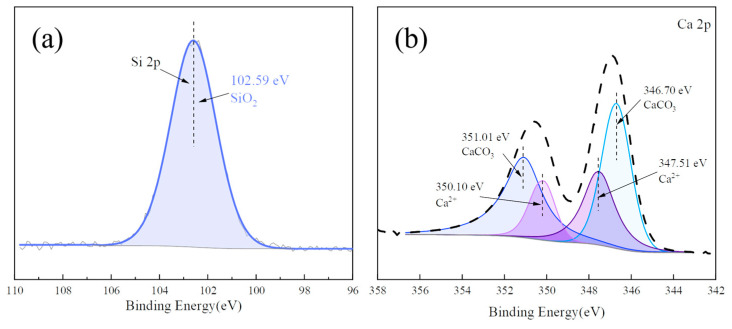
Si 2p (**a**) and Ca 2p (**b**) XPS spectra of the CMS.

**Figure 8:**
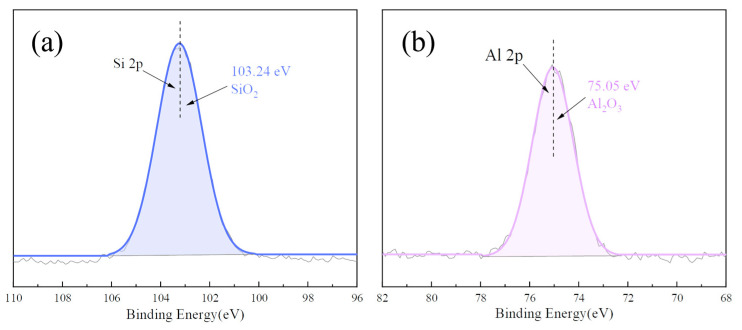
Si 2p (**a**) and Al 2p (**b**) XPS spectra of the HAFA.

The XPS spectrum of the main elements (Si, Ca) in the CMS was analyzed and the results are shown in Figure 7. For Si 2p, only quartz (SiO_2_) was confirmed using XPS with a binding energy of 102.59 eV. The existence of CaCO_3_ and Ca^2+^ in the CMS was observed on spectrum of Ca2p with the main peaks at 346.70 eV and 347.51 eV, which were assigned to calcite and andradite [40]. Figure 8 presents the XPS spectrum of the main elements (Si, Al) in the HAFA, where Si was essentially in the form of mullite (SiO_2_) with a binding energy of 103.24 eV, and the peak of 75.05 eV in Al 2p corresponded to Al_2_O_3_.

**Figure 9:**
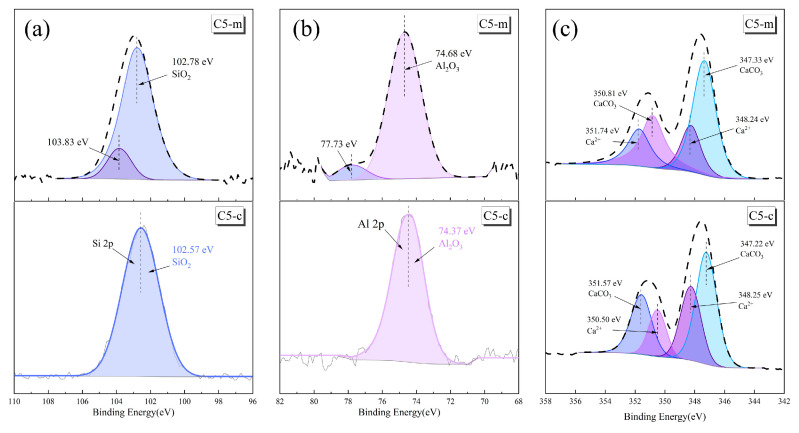
Si 2p (**a**), Al 2p (**b**), and Ca 2p (**c**) XPS spectra of the materials under a rotation speed of 600 rpm.

Sample C5 was selected for its best mechanical property and the results of the XPS analysis of the main elements (Si, Al, Ca) before and after curing are shown in Figure 9. Figure 9a illustrates the XPS spectrum of Si 2p, where the characteristic peak of C5-c was basically the same as the two raw materials, with only the peak of 102.57 eV corresponding to SiO_2_. The characteristic peak of SiO_2_ in C5-m was slightly weaker, while a novel characteristic peak with a binding energy of 103.83 eV emerged in the spectrum. This was the consequence of a conversion of the silicate structure into silica-active monomers during mechanical ball milling, and the repolymerization of these active monomers to form new silica-containing compounds during the curing process, hence the appearance of a new characteristic peak in the XPS spectrum. Figure 9b presents the XPS spectrum of Al 2p with binding energies of 74.37 eV and 74.68 eV for Al_2_O_3_ in C5-c and C5-m, respectively, which was slightly lower compared to the HAFA spectrum (Figure 8). The binding energy reduction in SiO_2_ and Al_2_O_3_ in the cured specimens was a demonstration of the reconstitution of the Si–O and Al–O bonds into a new silica–aluminate system after breaking. A new characteristic peak with a binding energy of 77.73 eV appeared in C5-m, resulting from the polymerization of a great amount of aluminous monomer or reactive groups produced during mechanical ball milling, corresponding to the reconfiguration of the Al–O bond in the cured specimens [41]. Figure 9c illustrates the XPS spectrum of Ca 2p with peaks of 347.22 eV and 347.33 eV for CaCO_3_ in C5-c and C5-m, respectively. The increase in the binding energy compared to the CMS (Figure 7) indicated a chemical bond breakage of CaCO_3_ in the cured specimens [42].

### 3.3. Toxic Leaching Procedure

As summarized in Table 5, the concentrations of the heavy metals in both raw materials were within their limits, except for a slight exceedance of Mn in the CMS and As in the HAFA. Therefore, only part of the cured body samples were subsequently selected for leaching toxicity testing. After 28 days of curing, the concentration of the main heavy metals in the cured specimens were all reduced below the standard limits and the final cured products were toxicity free.

## 4. Conclusions

A compounded cementitious material with copper smelting slag and high-aluminum fly ash as the main raw materials was successfully prepared with a 28-d compressive strength of up to 31.22 MPa. The heavy metal leaching toxicity was below the limits of the relevant standards GB 3838-2002 and GB/T 14848-2017.

(1) Based on mechanical ball milling, the activation of the mineral cementing activity of CMS and HAFA was achieved. While improving the particle size gradation of the raw materials, the initial activation was obtained by altering the physicochemical properties through the selection of a suitable ball milling speed. Gels were generated during the subsequent hydration process, and a large amount of cementing material wrapped around the aggregates to form a morphologically stable and tightly structured gel system, which improved the mechanical strength of the cured products.
(2) The CMS and HAFA particles were grounded easily and the D50 was reduced to 4.04 μm after 1 h of ball milling at the optimum rotation speed (600 rpm). The optimum material proportion was 1:4. The addition of fine particles of HAFA filled the voids in a coarse aggregate and simultaneously produced a microaggregate effect, which improved the denseness of the cementitious material, but excessive content disrupted the balance of coarse and fine particle combination, thus affecting the structural strength and mechanical properties of the materials.
(3) Heavy metal contaminants were kept at a relatively low content and low toxicity throughout the process from the raw materials to the preparation of the cementitious materials, which was environmentally friendly and safe to operate. Based on leaching tests, the mobility of heavy metal pollutants in the curing block was considered low, the leaching concentration of heavy metals was below the relevant standard limits, and the prepared cementitious materials could be utilized in scenarios such as road foundations, lagoon closures, and quarry restoration.

CMS and HAFA could be mechanically and chemically activated in synergy to produce cementitious materials with excellent compressive strengths. The preparation process was eco-friendly and low in carbon, simple to operate, and offered a high stability of heavy metals, which was suitable for application in practical environmental remediation projects and could provide effective solutions for ecological environment construction. Further research on how to improve the utilization rate of copper smelting slag and investigations about the leaching behavior under different conditions of the proposed cementitious materials is worth pursuing.

## Figures and Tables

**Table 1:** Mixture proportions and properties of the cementitious materials.

Effect Factor	Number	CMS: HAFA	Rotation Speed	CaO Content
Material proportion	A1	1:1	550	5%
	A2	1:2	550	5%
	A3	1:3	550	5%
	A4	1:4	550	5%
	A5	1:5	550	5%
	A6	1:6	550	5%
CaO dosage	B1	1:4	550	3%
	B2(A4)	1:4	550	5%
	B3	1:4	550	7%
	B4	1:4	550	9%
	B5	1:4	550	11%
	B6	1:4	550	13%
Rotation speed	C1	1:4	350	5%
	C2	1:4	450	5%
	C3	1:4	500	5%
	C4(A4)	1:4	550	5%
	C5	1:4	600	5%
	C6	1:4	650	5%

**Table 2:** Major particle size of the raw materials and blended mill products.

Sample	CMS	HAFA	C1	C2	C3	C4	C5	C6
D_50_/μm	97.57	22.26	10.04	6.66	4.93	3.50	4.04	3.33
D_90_/μm	258.35	62.46	32.50	23.10	18.38	14.29	16.36	15.32

**Table 3:** Main chemical composition of the raw materials (%).

Materials	CaO	SiO_2_	Al_2_O_3_	Fe_2_O_3_	MgO	SO_3_
CMS	30.51	29.12	2.97	26.00	6.41	3.08
HAFA	4.69	44.60	38.47	3.73	0.58	3.80

**Table 4:** Concentration of the trace elements (ppm).

Materials	Mn	Ti	Cu	Pb	Zn	Ag	Cr	Ni
CMS	6860	2720	942	n *	375	199	67	60
HAFA	607	35,200	309	480	671	n	231	154

n * = not detected.

**Table 5:** Leaching concentration of the heavy metals (mg/L).

Elements	Cr	Mn	Cu	Zn	Pb	As	Cd
CMS	0	4.569	0.212	0.067	0	0.042	0
HAFA	0	0.987	0.014	0.646	0	0.127	0.006
C1	0	0.069	0.003	0.008	0	0.022	0
C2	0	0.003	0	0.009	0	0.035	0
C3	0	0.237	0.004	0.017	0	0.018	0
C4	0	0.184	0.003	0.017	0	0.016	0
C5	0	0.275	0.002	0.012	0	0.014	0
C6	0	0.485	0.002	0.017	0	0	0
GB 3838-2002	0.05	n	1.0	2.0	0.05	0.1	0.005
GB/T 14848-2017	0.1	1.5	1.5	5.0	0.1	0.05	0.01

## Data Availability

Data are contained within the article.
